# Genomic Tools in Groundnut Breeding Program: Status and Perspectives

**DOI:** 10.3389/fpls.2016.00289

**Published:** 2016-03-17

**Authors:** P. Janila, Murali T. Variath, Manish K. Pandey, Haile Desmae, Babu N. Motagi, Patrick Okori, Surendra S. Manohar, A. L. Rathnakumar, T. Radhakrishnan, Boshou Liao, Rajeev K. Varshney

**Affiliations:** ^1^International Crops Research Institute for Semi-Arid TropicsPatancheru, India; ^2^International Crops Research Institute for Semi-Arid TropicsBamako, Mali; ^3^International Crops Research Institute for Semi-Arid TropicsKano, Nigeria; ^4^International Crops Research Institute for Semi-Arid TropicsLilongwe, Malawi; ^5^Directorate of Groundnut ResearchJunagadh, India; ^6^Oil Crops Research Institute Chinese Academy of Agricultural SciencesWuhan, China

**Keywords:** groundnut, breeding, selection, genomics, markers, varieties

## Abstract

Groundnut, a nutrient-rich food legume, is cultivated world over. It is valued for its good quality cooking oil, energy and protein rich food, and nutrient-rich fodder. Globally, groundnut improvement programs have developed varieties to meet the preferences of farmers, traders, processors, and consumers. Enhanced yield, tolerance to biotic and abiotic stresses and quality parameters have been the target traits. Spurt in genetic information of groundnut was facilitated by development of molecular markers, genetic, and physical maps, generation of expressed sequence tags (EST), discovery of genes, and identification of quantitative trait loci (QTL) for some important biotic and abiotic stresses and quality traits. The first groundnut variety developed using marker assisted breeding (MAB) was registered in 2003. Since then, USA, China, Japan, and India have begun to use genomic tools in routine groundnut improvement programs. Introgression lines that combine foliar fungal disease resistance and early maturity were developed using MAB. Establishment of marker-trait associations (MTA) paved way to integrate genomic tools in groundnut breeding for accelerated genetic gain. Genomic Selection (GS) tools are employed to improve drought tolerance and pod yield, governed by several minor effect QTLs. Draft genome sequence and low cost genotyping tools such as genotyping by sequencing (GBS) are expected to accelerate use of genomic tools to enhance genetic gains for target traits in groundnut.

## Introduction

Groundnut (*Arachis hypogaea* L.) or peanut, an important oilseed and food legume crop, is cultivated in 25.44 million ha world over with a total production of 45.22 million tons during 2013 (Food and Agriculture Organization of United Nations, 2014). China and India are the leading groundnut producers followed by USA and Nigeria. Africa with 12.40 m ha area and 11.54 m tons of production, and Asia with 11.87 m ha and 29.95 m tons, together account for 95% global groundnut area and 91% of global groundnut production. There was a substantial increase in global groundnut production by about 5 m tons in 2013, from 40 m tons in 2012. In the last decade, 2004–2013, global groundnut production increased by 24%, contributed by 7% increase in groundnut area, and 16% increase in yield. The projected global demand for groundnut and its related products is expected to increase and so there is a need to further increase production and productivity to meet the demand.

Groundnut is ranked fifth among oilseed crops in the world after oil palm, soybean, rapeseed, and sunflower (Food and Agriculture Organization of United Nations, 2013). Traditionally, groundnut has been used for extraction of oil for edible and industrial purposes, however its use as food has been equally important. In Europe, North and Central America, more than 75% of the available supply is used as food. In Africa it is used for both food and oil for cooking purposes. Within Asia—India, China, Indonesia, Myanmar, and Vietnam use groundnut oil for cooking purpose, while in Thailand it is consumed as food. Globally, 41% of groundnut produced is used for food purposes and 49% is crushed for extraction of oil. The oilcake meal remaining after oil extraction is used as industrial raw material and also as a protein supplement in livestock feed rations. However, the use of groundnut as direct and processed food products has been expanding in countries like China and India. Groundnut kernels can be consumed directly as ready-to-eat products or indirectly as confectionary. The inferior quality oil is used for making soaps, detergents, cosmetics, paints, candles, and lubricants. The groundnut shells are used as fuel source, as filler for making particle boards and as animal feed. It can also be used as bedding material for poultry or as mulching material to reduce evaporative losses during summer season. Groundnut is both a cash crop and healthy food crop contributing to nutrition of farm families and households, and is also a source of nutritious fodder (haulms) for livestock.

Groundnut kernels contain 40–60% oil, 20–40% protein, and 10–20% carbohydrates. They provide 567 kcal of energy from 100 g of kernels (USDA nutrient database). Additionally, they also contain several health enhancing nutrients such as minerals, antioxidants, and vitamins. They contain antioxidants like *p*-coumaric acid and resveratrol, Vitamin E, and many important B-complex groups of thiamin, pantothenic acid, vitamin B-6, foliates, and niacin. Groundnut is a dietary source of biologically active polyphenols, flavonoids, and isoflavones. As they are highly nutritious, groundnut and groundnut based products are promoted as nutritional foods to combat energy, protein, and micronutrient malnutrition among the poor. Groundnut in the form of flour, protein isolates and meal in a mixed product is very desirable. In countries like Africa where malnutrition is a major problem, groundnut based ready-to-use-therapeutic food products like “Plumpy nut” and peanut butter has helped to save the lives of thousands of malnourished children (UNICEF, 2007).

The groundnut haulms contain 8–15% protein, 1–3% lipids, 9–17% minerals, and 38–45% carbohydrates. It is used as cattle feed either in fresh or dried stage, or by preparing hay or silage. The digestibility of nutrients in groundnut haulm is around 53% and that of crude protein is 88% when fed to cattle. Haulms release energy up to 2.337 cal kg^−1^ of dry matter. Being a legume crop, groundnut has unique ability to fix atmospheric nitrogen through symbiotic association with *Rhizobium*. This ability is greatly useful in improving the fertility of degraded soils, and economically beneficial to the small-holder farmers as they can save money on resources such as nitrogen fertilizers.

Improved groundnut varieties resulting from genetic improvement have contributed to enhanced production and productivity, and meet the needs of the producers, processors, and consumers. The yield productivity increase varied across different growing regions. Wide range of varieties of groundnut are cultivated to meet the food, oil, and industrial needs. Groundnut breeding programs have extensively used phenotyping tools for selecting plants/progenies with desirable traits (Janila et al., 2013). The conventional breeding procedures employ hybridization, phenotype based selection followed by selection of promising breeding lines through yield evaluation trials. With the advent of genomic tools, marker assisted breeding (MAB) was deployed to enhance efficiency of selection of target traits in groundnut (Pandey et al., 2012; Janila et al., 2013; Varshney et al., 2013). Moreover, genomic tools enable to combine several important target traits in a single variety. The purpose of this review is to present an overview of deployment of genomics assisted breeding, and recent developments in the integration of modern genomic tools into the conventional breeding framework to develop improved groundnut varieties. Besides, groundnut breeding through conventional plant breeding methods is briefly discussed to appraise the progress made so far and identify the areas where conventional breeding methods can be complemented with genomic tools to achieve higher rate of genetic gain for target traits.

## Taxonomy of *Arachis*

Cultivated groundnut (*A. hypogaea* L.) is self-pollinated, allotetraploid (2*n* = 4*x* = 40) with a genome size of 2891 Mbp. The genus *Arachis* belongs to the family Fabaceae, subfamily Faboideae. Based on morphology, geographical distribution, and cross compatibility, the genus is divided into nine taxonomic sections. The genus comprises of 80 described species (Valls and Simpson, 2005), that include diploids and tetraploids, of which *A. hypogaea* L. is most important and widely cultivated. *A. hypogaea* is a segmental amphidiploid but cytologically behaves like a diploid. Based on growth habit and flower arrangement on the main axis, cultivated groundnut is divided into two subspecies subsp. *fastigiata* and subsp. *hypogaea*. The *fastigiata* subsp is comprised of four botanical varieties, var. *vulgaris*, var. *fastigiata*, var. *peruviana*, and var. *aequatoriana*, while *hypogaea* is divided into two varieties, var. *hypogaea* and var. *hirsuta* based on inflorescence, pod, and seed characteristics. There are three market types, Virginia, Spanish, and Valencia. *A. hypogaea* subsp. *fastigiata* var. *vulgaris* are Spanish bunch types, *A. hypogaea* subsp. *hypogaea* var. *hypogaea* is Virginia market type, and *A. hypogaea* subsp. *fastigiata* var. *fastigaita* is Valencia market type. Cytological and molecular studies suggest that two diploid wild species *Arachis duranensis* (AA) and *Arachis ipaensis* (BB) are progenitors of cultivated groundnut. A single hybridization event between progenitors followed by chromosome duplication about 3500 years ago led to origin of cultivated groundnut. Based on cross-compatibility among the members of the genus *Arachis*, four gene pools were identified (Singh and Simpson, 1994).

## Genetic resources

The gene bank at ICRISAT, India holds the largest collection of groundnut accessions. The other gene banks with groundnut collection are, National Bureau of Plant Genetic Resources (NBPGR) and Directorate of Groundnut Research (DGR) in India; Oil Crops Research Institute (OCRI) of Chinese Academy of Agricultural Sciences (CAAS) and Crops Research Institute of Guangdong Academy of Agricultural Sciences in China; U. S. Department of Agriculture—Agricultural Research Service, Tifton Georgia; Texas A&M University; and North Carolina State University in the USA; EMBRAPA—CENARGEN and the Instituto Agronômico de Campinas in Brazil; and Instituto Nacional de Tecnología Agropecuaria (INTA) and Instituto de Botánica del Nordeste (IBONE) in Argentina. The number of groundnut accessions available in the gene banks has been reviewed by Pandey et al. (2012). For the ease in identification of trait-specific genetic resources, core and mini core collections that represent 10 and 1% of the entire germplasm collection, respectively, were developed at ICRISAT, USDA/ARS, and China. The gene banks are also repositories of wild species which are source of novel alleles.

## Target traits in groundnut breeding

A wide array of traits has been targeted for genetic improvement in groundnut by plant breeders, and the choice of the trait varies from region to region depending on growing seasons, producer needs, consumer demands, market preferences, and industrial requirements. In US and several other European countries where groundnut is mostly consumed as food and the average productivity is high, focus has also been on developing varieties with improved food quality and flavor traits, and freedom from mycotoxins and allergens in the production. In countries of Asia and Africa where yield is low, the focus is to improve yield by plugging the yield gap through genetic tolerance to biotic and abiotic stress factors. The important yield contributing parameters in groundnut that are targeted for improvement include: pod yield per plant, number of pods per plant, shelling percentage, sound mature kernel percentile, and 100 seed weight. Besides, several indirect factors such as ease in harvesting (peg strength) and shelling, number of seeds per pod, reticulation, beak and constriction of pods, kernel shape and color, fresh seed dormancy, and blanching ability were also considered to satisfy farmers', processors', and market demands. Fresh seed dormancy is important especially in Spanish bunch types which are prone to pre-harvest sprouting. Pre-harvest sprouting can cause 10–20% yield loss (Nautiyal et al., 2001) and predisposes the produce to the attack of fungus and microbes. A short period of dormancy of about 10–15 days is desirable.

Foliar fungal diseases, late leaf spot (LLS) caused by *Phaeoisariopsis personata* (Berk. and M.A. Curtis) van Arx., early leaf spot (ELS) caused by *Cercospora arachidicola* Hori and rust caused by *Puccinia arachidis* Spegazzini are major production constraints of groundnut crop globally. Stem and pod rot caused by *Sclerotium rolfsii* is emerging as a major threat in several growing regions. Bacterial wilt caused by *Ralstonia solanacearum* is predominant among bacterial diseases of groundnut in South-East Asia, particularly China. Groundnut rosette disease (GRD) in Africa, Peanut Bud Necrosis Disease (PBND) in India, tomato spotted wilt virus (TSWV) in East and South East Asia, peanut stem necrosis disease (PSND) in some areas in Southern India, and peanut clump virus disease (PCVD) in West Africa are economically important viral diseases. Among nematodes, peanut root-knot nematode (*Meloidogyne* spp.), and the root-lesion nematode (*Pratylenchus brachyrus*) can cause significant economic losses in many groundnut production areas of the world. Resistance/tolerance to fungal/bacterial/virus/nematode diseases is important to plug the yield gaps of different production environments. The colonization of groundnut kernels by *Aspergillus* sp. (*A. flavus* and *A. parasiticus*) is a major constraint affecting groundnut quality globally. Infection to seeds can occur at pre-harvest or during post-harvest storage and colonization is followed by release of aflatoxin, a potent carcinogen. Several countries have strict regimes on permissible limit of aflatoxins in their imports on groundnut. Freedom from aflatoxin contamination is important for food safety and has trade implications (Janila et al., 2013).

Aphids (*Aphis craccivora* Koch), several species of thrips (*Frankliniella schultzei, Thrips palmi*, and *F. fusca*), leaf miner (*Aproaerema modicella*), red hairy caterpillar (*Amsacta albistriga*), jassids (*Empoasca kerri* and *E. fabae*), and Spodoptera are important foliar insect pests and cause localized damage to groundnut during different growth stages. Aphids and thrips are also vectors of important virus diseases. Termites, white grubs, and storage pests also cause damage to groundnuts. Among storage pests, groundnut borer or weevil (*Caryedon serratus*) and rust-red flour beetle (*Tribolium castaneum*) are important.

Most of the groundnut cultivation occurs in the semi-arid regions where water is often a limiting factor. Mid- and end-of-season drought are critical as they directly affect pod yield and quality. Development of water use efficient cultivars has been an important target trait. Breeding for short duration groundnut is an escape mechanism to avoid end-of season moisture stress. Breeding for heat resilient crops has been gaining wide attention as heat tolerant genotypes can sustain production in heat stress environments that are expected to increase as a consequence of climate change. It is possible that moisture and temperature stress together may have adverse effects on productivity of groundnut in its semi-arid production environment. With regards to the ability of groundnut to fix atmospheric nitrogen, variability is known at the level of both, host-plant and microbe (Janila et al., 2013). However, pod yield is considered as an indirect measure of nitrogen fixation ability for selection in groundnut.

In Asia (India and China) and Africa, where groundnut is used for oil, improving oil content is an important target trait. Worldwide demand of groundnut for direct food consumption has been steadily increasing, with the developing countries accounting for much of this increase. In Asia, with change in trend toward food uses, quality parameters to suit the food, and industry needs have emerged as important target traits in several countries including India and China. In the case of roasted peanut the flavor is an essential characteristic influencing consumer acceptance, and enhancing roasted peanut flavor is an important target trait. The quality attributes preferred for confectionary grade varieties include high protein and sugar, low oil and aflatoxin contamination, attractive seed size and shape, pink or tan seed color, and ease of blanching and high oleic/linoleic fatty acid (O/L) ratio (Dwivedi and Nigam, 1995). Blanchability, removal of testa or seed coat (skin) from raw or roasted groundnuts is of economic importance in processed groundnut food products, which include peanut butter, salted groundnuts, candies, and bakery products and groundnut flour. High oleic trait is important for consumers' health and for food industry. Groundnut based food products are now widely used in feeding programs to treat malnutrition and therefore, improving nutritional quality traits is gaining importance. Nutritional quality adds value to ready-to-use therapeutic food (RUTF) and other food supplement for children and elderly. For fodder purpose, the nitrogen content, metabolizable energy, and organic matter digestibility of haulms are important quality traits. Depending on breeding objective one or more traits may be given preference over others. Both, nutritional quality traits and food processing quality traits are gaining importance in the breeding programs to meet various uses as well as consumer preference.

## Groundnut improvement using conventional breeding methods

Crop improvement via conventional breeding makes use of suitable breeding method to screen, identify and select superior performing line(s) in a set of genotypes or in a population developed by crossing diverse individuals. The breeding method to be used depends on the target trait, genetic nature of the trait, and resource availability. Breeding methods used for groundnut using single or multiple crossing systems have been reviewed by Janila and Nigam (2012). Studies on inheritance, heritability and trait associations of target traits have been useful in breeding programs. Heritability for seed yield, drought tolerance and its component traits was reported to be low to high in different populations. While for 100 seed/pod weight, and for days to flowering and maturity, high heritability was reported. Pod yield in groundnut is positively associated with number of mature pods per plant and 100 kernel weight. SPAD (soil plant analysis development) chlorophyll meter is used in the trait-based groundnut breeding for drought tolerance. Disease score for LLS is associated with component traits of resistance consequently, disease scores in the field suffice to evaluate the breeding material for resistance to LLS. Absence of trade-off between oil content and yield (Janila et al., 2014) enabled development of high oil yielding breeding lines. Back cross breeding was rarely used because most of the desired economic traits in groundnut are quantitatively inherited. However, with the advent of molecular markers for the traits of interest, back cross breeding has been used to transfer major effect QTLs governing resistance to nematode and foliar fungal diseases, and mutant FAD (Fatty acid dehydrogenase) alleles for enhanced fatty acid profile. Wide hybridization techniques were used to tap the potential of wild species, particularly for disease resistance, while mutation breeding played an important role for release of several groundnut varieties in India and China.

Most of the approaches centered on pod yield improvement and tolerance/resistance to diseases and drought. Pod yield under stress conditions, such as, diseases and drought is measured and used as a selection criteria in breeding. However, with the development of efficient phenotyping tools such as, near infrared reflectance spectroscopy (NIRS) and nuclear magnetic resonance (NMR) spectroscopy, and tools for screening disease resistance using disease scores, and water use efficiency (WUE) through surrogate traits the focus has shifted to trait-based breeding (Janila and Nigam, 2012). By 2012, the national programs in India have released 194 improved groundnut varieties for cultivation in the country following the conventional breeding methods (Rathnakumar et al., 2013). Below we reviewed trait improvement achieved through conventional breeding in groundnut, while critically examining the gaps to achieve enhanced genetic gains for these target traits. Some trait-specific commercial groundnut varieties released for cultivation are listed in Table 1.

**Table 1:** **Some examples of commercial groundnut lines released for different target traits through conventional breeding approaches**.

**Trait (s)**	**Variety/Line/Genotype**	**References**
**AGRONOMIC TRAITS**		
Early-maturity	JL 24 (Phule Pragati), Nyanda	Patil et al., 1980; Upadhyaya et al., 2005
Medium-maturity	Somnath	Badigannavar and Mondal, 2007
High pod yield	TAG 24, TG 26, SB XI, GAUG 1	Patil et al., 1980; Badigannavar et al., 2002
Wide adaptability	JL 24 (Phule Pragati) and TG 37A	Kale et al., 2004
**RESISTANCE TO DISEASES AND INSECT PESTS**		
Root-Knot Nematode (*Meloidogyne* spp.)	COAN	Simpson and Starr, 2001
Kalahasty Malady	Tirupati-3	Mehan et al., 1993
LLS and rust	GPBD 4	Gowda et al., 2002
Peanut bud necrosis disease	Kadiri 3, ICGS 11, ICGS 44, ICGS (FDRS) 10, ICGV 86325, DRG 17, CSMG 884	Ghewande et al., 2002
Tomato spotted wilt virus	Florida 07, C-99R, Florida Runner, UF 91108	Branch, 1994, 1996; Culbreath et al., 1997; Gorbet and Tillman, 2009
Groundnut rosette disease (GRD)	Samnut 24 (ICIAR19BT), Samnut 25 (ICGX-SM 00020/5/10), and Samnut (26ICGX-SM 00018/5/P15/P2)	Ajeigbe et al., 2015
Bacterial wilt disease	Zhonghua 4, Zhonghua 6, Tianfu 11, Zhonghua 21	Yu et al., 2011
Low aflatoxin contamination	J-11, 55-437, ICG 7633, ICG 4749, ICG 1326, ICG 3263, ICG 9407, ICG 10094, ICG 1859, ICG 9610	Nigam et al., 2009
**ABIOTIC STRESS TOLERANCE**		
Drought tolerance	ICGV 91114, ICGV 87846, ICR 48, ICGV 00350, 55-437, GC 8-35, 55-21, 55-33, SRV 1-3, SRV 1-96	Mayeux et al., 2003; Vindhiyavarman et al., 2014
Heat stress	55-437, 796, ICG 1236, ICGV 86021, ICGV 87281, ICGV 92121	Craufurd et al., 2003
**QUALITY AND NUTRITIONAL PARAMETERS**		
High oleic acid content	SunOleic 95R, SunOleic 97R	Gorbet and Knauft, 1997
High oil content	ICGV 03057, ICGV 03042, ICGV 05155, ICGV 06420, ICGV 03043	Annonymous, 2014
Iron and zinc content	ICGV 06099, ICGV 06040	Janila et al., 2014
**CONFECTIONARY TRAITS**		
Large seeded pods	ICGV 03137, Asha, Mallika, SC Orion, TG 1, TKG 19A, Somnath, TPG 41, TLG 45, TG 39	Hildebrand and Nosenga, 2005; Badigannavar and Mondal, 2007; Janila et al., 2012
**BREEDING FOR MULTIPLE TRAITS**		
Flavor quality and high yield	FLORUNNER	Norden et al., 1969
Medium-maturity and GRD resistance	Samnut 21, Samnut 22, Samnut 23	Ajeigbe et al., 2015
Early-maturity and GRD resistance	Samnut 24, Samnut 25, Samnut 26	Ajeigbe et al., 2015
Multiple disease and insect resistance	ICGV 86699	Reddy et al., 1996
High O/L ratio and TSWV resistance	Hull	Gorbet, 2007
High oleic acid and moderate resistance to TSWV, stem rot and Sclerotina blight	Tamrun OL01	Simpson et al., 2003b

### Improvement of yield and yield related traits

Groundnut yield can be classified based on utility into pod, kernel, oil, and haulm yield. Pod yield is most important and is a function of crop growth rate, duration of reproductive growth, and the fraction of crop growth rate partitioned toward pod yield (Janila et al., 2013). Selection for yield were used for improving groundnut productivity but the genetic gains from such selection were often hampered by the complex nature of the trait and large G × E interaction effects (Nigam et al., 1991). Majority of the efforts toward increasing yield in India came from improvement in seed size, seed weight, and number of pods per plant (Rathnakumar et al., 2012). It was reported that improved varieties alone contributed to 30% yield increase in India since 1967 (Reddy and Basu, 1989). JL 24, a high yielding variety with wide adaptability has been release in several countries. It was released as Phule Pragati in 1979 in India (Patil et al., 1980), subsequently, it was introduced to Africa and released as JL 24 in Congo (1990), Sera Leone (1993), and South Africa (2002), as Luena in Zambia (1999), as Kakoma in Malawi (2000), as Saméké in Mali (2000), as ICG 7827 in Mozambique (2011) and is commercially cultivated in several other countries (Chiyembekeza et al., 2001). It was also released in 1985 as Sinpadetha 2 in Myanmar and in 1992 as UPL Pn 10 in the Philippines.

### Breeding for biotic and abiotic stress tolerance/resistance

Improved breeding lines with resistance to foliar fungal diseases were developed (Singh et al., 2003), and “Southern Runner” was the first moderate LLS resistant cultivar to be released in the USA (Gorbet et al., 1987). As compared to the cultivated *A. hypogaea*, high levels of resistance to foliar fungal diseases were reported in wild species (Stalker and Simpson, 1995) and they were utilized to derive interspecific hybrids, which in turn were used to develop varieties, such as, GPBD 4 (Gowda et al., 2002) and Mutant (28-2) (Motagi et al., 2014). ICG 7878, released in Mali is resistant to ELS and LLS. The foliar fungal disease resistant varieties developed in 1980's and 1990's such had poor pod and kernel features due to linkage disequilibrium, consequently, despite high pod yield and resistance, they did not find acceptance among farmers (Nigam, 2000). Combining foliar fungal disease resistance and early maturity has remained a challenge despite availability of several donors.

The development of simple and efficient field screening techniques formed the basis of breeding for resistance to PBND, TSWV, and GRD. Varieties tolerant to PBND, TSWV, and GRD were released for cultivation (Table 1). Resistance sources to GRD were first discovered in Senegal in 1952 and used as parents in breeding high-yielding and rosette-resistant groundnut varieties such as, RMP12, RMP 91, and RG1. However, most of these varieties were late maturing, consequently not preferred by farmers (Waliyar et al., 2007). ICGV SM 90704, released in Uganda as Igola 2 is a high-yielding, long-duration variety with resistance to rosette. A short duration Spanish bunch variety, ICG 12991 is also resistant to rosette. In Nigeria, medium duration and GRD resistant varieties *viz*., UGA 2 (Samnut 21), M 572.80I (Samnut 22), and ICGV-IS 96894 (Samnut 23) were released in 2001, and more recently three early maturing GRD resistant varieties Samnut 24, Samnut 25, and Samnut 26 were released. Studies have shown that these varieties have minimized the incidence of GRD in Nigeria (Ajeigbe et al., 2015). ICGV-SM 08503, ICGV-SM 08501, ICGV-SM 01731, ICGV-SM 01724 ICGV-SM 01514, ICGV-SM 99551, and ICGV-SM 99556 are GRD resistant varieties released for cultivation in Malawi during 2014.

The resistance gene to *Meloidogyne arenaria* was introgressed into *A. hypogaea* by using a complex interspecific hybrid comprising of three nematode resistant species, *Arachis batizocoi, Arachis cardenasii*, and *Arachis diogoi* Hoehne and the first resistant cultivar TxAG-6 was released for commercial cultivation (Simpson et al., 1993). This was followed by the release of TxAG-7, derived from backcross (Simpson et al., 1993). Efficient screening methods for resistance to nematodes enabled identification of resistant source, and subsequently used to breed genotypes with resistance to root-knot nematodes (Simpson et al., 2003a) and Kalahasti malady (*Tylenchorhynchus brevilineatus*) (Mehan et al., 1993). Tifguard is a groundnut variety bred for resistance to both root-knot nematode and TSWV and was released for cultivation in the USA (Holbrook et al., 2008). Although, varieties with multiple resistance are needed, simultaneous targeting of multiple diseases is laborious with conventional breeding approaches, consequently resistance to one disease is often targeted.

Bacterial wilt resistant sources in cultivated (Liao et al., 2005) and wild *Arachis* species (Tang and Zhou, 2000) were used to develop and release resistant groundnut cultivars, Zhonghua 4, Zhonghua 6, Tianfu 11, Zhonghua 21, etc., in China (Yu et al., 2011) and other countries. Resistant sources to pre-harvest seed infection, *in-vitro* seed colonization (IVSC) and aflatoxin production by *A. flavus* were identified in cultivated groundnut using *in-vitro* and *in-situ* colonization techniques (Mehan et al., 1991). Sources with resistance operating at three different levels, pod wall, testa, and cotyledons were reported. Despite reports on availability of sources of resistance, progress in breeding is limited by lack of reliable screening protocols. Both, *in-vivo* and *in-vitro* techniques suffer from repeatability and reliability. In groundnut breeding programs across the world, breeding for resistance to diseases has received more attention than breeding for resistance to insect pest except when they are vector of viral disease. Another important reason for this is the non-availability of the resistant sources for insects in cultivated and wild *Arachis* species. Limited progress has been made in screening of improved varieties for storage pest tolerance although variability has been reported.

Significant progress has been achieved in understanding the underlying mechanism of drought tolerance in groundnut over the years which has resulted in development of efficient physiological trait-based and empirical selection approaches (Nigam et al., 2005) to breed for drought tolerance in groundnut. Empirical approach that measured yield under water limiting conditions is widely used. Trait-based approaches measuring WUE employs SPAD (soil plant analysis development) and SLA (specific leaf area) for drought tolerance, and they are often used in combination with empirical approach. Root traits are identified as drought adaptive traits, however their use as selection criteria for drought resistance is limited as they required elaborate phenotyping protocols. So far, studies on heat tolerance in groundnut were limited to few screening studies reporting tolerant lines for heat stress (Craufurd et al., 2003; Hamidou et al., 2013).

### Breeding for quality traits

The quality attributes preferred for confectionary grade varieties such as high sugar, low oil, and free from aflatoxin contamination, attractive seed size and shape, pink or tan seed color, and ease of blanching and high oleic/linoleic (O/L) ratio were targeted for improvement (Nigam et al., 1989; Dwivedi and Nigam, 1995). With the development of tools for non-destructive analysis of samples breeding for enhanced oil content and quality has become possible. It is now possible to screen whole kernels and even single seeds using techniques like NIRS and NMR spectroscopy. Following hybridization and wide scale screening efforts several high oil lines (>50%) were identified, however under field cultivations the stability was found wanting. Extensive multi-location testing identified four high oil yielding lines ICGV 05155, ICGV 06420, ICGV 03042, and ICGV 03043 for release in India. Breeding for high oleic groundnut began with the discovery of F435 a high oleic acid spontaneous mutant with an oleic acid content of >80% (Norden et al., 1987), and the first high oleic variety, SunOleic 95R with 82% oleic acid content was registered in 1997 (Gorbet and Knauft, 1997). ICGV 03137, a Virginia bunch variety with high blanchability (Janila et al., 2012) and ICGV 06099 and ICGV 06040 with high kernel Fe and Zn (Janila et al., 2014) were reported. Large seeded varieties, preferred for table purposes, such as Asha and Namnama were released in Philippines and Mallika was released in India.

## Genomic tools-breeding groundnut the genomics way

Knowledge of trait genetics combined with classical plant breeding methods and efficient phenotyping tools has been successful in the development and release of several improved cultivars with high yield potential, resistance to biotic and abiotic stresses, and enhanced/improved nutritional quality traits in groundnut. However, sometimes the efforts invested in classical breeding to improve a trait do not justify the final outcome, especially in the case of interspecific hybrids and traits that are quantitatively inherited and influenced by genotype × environment interaction effects. The technique of crossing diverse parents for trait(s) of interest and selecting progenies in segregating generations usually works for traits with high heritability and for which efficient phenotyping tools are available such as resistance to LLS and rust. The desirable alleles of a trait with high heritability will be fixed in the successive generations of selfing in the presence of selection pressure. Nonetheless, phenotyping for making selection, for example, screening in disease nursery, involves huge resources, and time, besides the chance selection of escapes. Moreover, screening of some traits have to be done in a specific season and/or location. Screening for bacterial wilt resistance is carried out in disease endemic locations. Foliar fungal diseases occur in rainy season in Asia and Africa and field screening is possible in that season. While in the other non-rainy season, generations are advanced based on yield and other attributes, consequently, the progress in breeding for disease resistance is often slow with low rate of genetic gains. Improvement of traits such as, oil content and quality through classical breeding was limited as they require efficient and robust phenotyping tools, and moreover for these traits phenotyping has to be done after harvest, drying, and shelling of the pods. Available tools for analyzing seed quality traits are destructive and can usually result in loss of valuable breeding material especially in early segregating generations where the seed material is in limited supply. Consequently, screening for quality traits is often delayed to advanced generations. Reliable and repeatable phenotyping requires practice and skill, a crucial aspect in breeding. However, even with the best available phenotyping tools, there is a possibility of selection bias that may occur due to chance failure of phenotypic screens and chance escapes. High throughput phenotyping tools are now available to screen for diverse traits, but their application is limited due to their high cost and lack of technical knowhow. Consequently, the high throughput phenotyping tools may not be ready for deployment in breeding programs, but they may be useful to establish marker-trait associations (MTA), genome-wide associations and for training genomic selection models which require precise phenotyping (Cobb et al., 2013). Genomic tools, on other hand offer cost-effective, robust, and reliable tools to enhance genetic gain for target traits and it is possible to enhance the efficiency of classical breeding by optimizing the time, resources, and funds.

The advent of genomic tools and their utilization in crop improvement programs has revolutionized the breeding methodologies. The deployment of genomic tools in groundnut improvement programs has begun recently. The slow progress can be in part attributed to tetraploid nature of groundnut, low marker polymorphism and lack of marker/genome sequence resources and high throughput genotyping platform. Among the approaches adopted to deploy genomic tools in groundnut improvement programs, marker assisted backcrossing (MABC) has been the most preferred and result oriented molecular breeding approach for improving existing popular genotypes for one or two traits and pyramiding of few genes/QTLs. MABC enables optimum utilization of time and resources, early selection of genotypes in segregating generations, break linkage disequilibrium associated with wild interspecific hybrids and carry out back cross breeding for trait improvement. Few success stories in groundnut improvement using MABC is described in the section “MAB for groundnut improvement.” Besides, marker assisted selection (MAS) has also been used in groundnut (Janila et al., 2016). However, in the case of quantitative traits such as drought resistance, yield etc., which are controlled by several QTLs, each with a small effect on the phenotype, it is very difficult to develop improved genotypes with ideal features through MABC (Varshney et al., 2013). In such situations, the other breeding approach namely genomic selection (GS) discussed under the section “emerging genomic technologies and groundnut genome sequence database” are being deployed in groundnut. As part to the Peanut Genomics Initiative, peanutbase.org, a resource database was developed for U.S. and international peanut researchers and breeders. The resources available includes, diagnostic markers for use in MAS, QTL information, maps of diploid and tetraploid *Arachis* species, diploid genome sequence data, and *A. hypogaea* transcriptome data.

### Mapping populations and marker-trait associations in groundnut

The application of genomic tools in groundnut breeding requires identification of genes/QTLs linked to traits of interest. The first step in this regard is development of mapping populations. Development of a mapping population by crossing genetically divergent parent(s) and using recent advances in marker technologies to fine map QTLs for economically important target traits has been used to establish MTA for deployment in groundnut breeding. In order to carry out marker trait association studies for agronomically important traits in groundnut, several types of genetic populations have been developed such as recombinant inbred lines (RILs), F_2_ population, near isogenic lines (NILs), backcross introgression lines (BILs), natural populations namely, groundnut reference set or minicore collection developed by ICRISAT, nested association mapping (NAM), and multi-parent advanced generation inter-cross (MAGIC) populations (Pandey et al., 2012; Varshney et al., 2013; Janila et al., 2014).

The trait mapping approaches can be broadly categorized into three types i.e., linkage mapping, linkage disequilibrium (LD) based genome-wide association studies (GWAS), and joint linkage-association mapping (JLAM). Trait mapping through linkage mapping in groundnut used bi-parental mapping populations which basically utilize the diversity present between two diverse parents for traits of interest through development of F_2_, BIL, RIL, and NIL populations. Initially the purpose of developing mapping populations was to map the maximum number of loci in a single map but later, populations were developed targeting mapping of economically important traits in groundnut such as tomato spotted wilt virus (TSWV), LLS and rust, drought related traits, GRD etc. The LD-based GWAS approach utilizes diverse germplasm sets with high variability for economically important traits in a crop species. In case of groundnut, two such efforts have been reported. The first attempt was made to conduct association analysis using US minicore collection which reported association between *FAD2* genes and oleic and linoleic acid trait (Wang et al., 2011). The second study used groundnut reference set developed by ICRISAT possessing global diversity i.e., comprising of 300 genotypes from 48 countries (Pandey et al., 2014). The later was most comprehensive GWAS study in groundnut as it targeted 50 agronomic traits and successfully reported identification of 524 highly significant MTAs for 36 traits with phenotypic variance ranging from 5.81% to as high as 90.09%. These MTAs with high PV, after validation could be used to improve biotic resistance, seed quality, drought tolerance related traits, and yield/yield components in groundnut (Pandey et al., 2014).

The third trait mapping approach i.e., JLAM requires development of multiple parent mapping population such as NAM and MAGIC. NAM makes use of both primitive and recent recombination events to take advantage of low marker density requirements, allele richness, high mapping resolution, and high statistical power. Whereas, MAGIC population utilizes multiple parents (8–12) from different origins and sometimes from exotic backgrounds in a crossing program so that multiple QTLs distributed across lines can be brought together into a single line through bi-parental matings. The availability of higher proportion of RILs from multiple parent crosses allows both coarse and fine mapping to be performed and the complex architecture of many traits which are associated with crop yield and quality can be deduced using epistatic interactions (Cavanagh et al., 2008). These two type of populations have not yet been used for trait mapping in groundnut. Nevertheless, the two NAM populations and three MAGIC populations are under development at ICRISAT in groundnut which will be later on used for conducting MTA studies. In addition, these two type of populations, in future, will serve as important resources for the discovery, isolation and transfer of essential genes to facilitate crop improvement. The detailed information on QTLs and the linked markers identified so far using linkage mapping approach is summarized in Table 2. A good number of studies reported QTLs for resistance to several diseases that include, rust, LLS, Bacterial wilt, nematode, *Sclerotinia minor, A. flavus*, Aflatoxin contamination, and TWWV. So far, major QTLs governing resistance to nematode, rust, and LLS were transferred through MABC. For drought tolerance several minor effect QTLs were reported.

**Table 2:** **Molecular markers associated with trait specific genes/QTLs in groundnut**.

**Population**	**Trait^#^**	Marker system^*^	**References**
Yuanza 9102 × ICGV 86699	Rust resistance	AFLP	Hou et al., 2007
*TAG 24 × GPBD 4*	Rust resistance	SSR	Khedikar et al., 2010
*A. duranensis* × *A. stenosperma*	LLS resistance	Legume anchor and resistance gene analog markers	Leal-Bertioli et al., 2009
*TAG 24 × GPBD 4, TG 26 × GPBD 4*	LLS and rust resistance	SSR	Sujay et al., 2012
Zhonghua 5 × J 11	Aflatoxin contamination	AFLP	Lei et al., 2005
Zhonghua 5 × J 11	Aflatoxin contamination	SCAR	Lei et al., 2006
12 genotypes	*Aspergillus flavus* resistance	SSR	Hong et al., 2009
*A. kuhlmannii* × *A. diogoi*	TSWV	AFLP	Milla et al., 2004
*Tifrunner × GT-C20, SunOleic 97R* × *NC94022*	TSWV	SSR	Qin et al., 2012
*ICG 12991 × ICGV-SM 93541*	Aphid (*Aphis craccivora*) resistance	AFLP	Herselman et al., 2004
21 Inter-specific and three cultivated lines	Peanut bud necrosis resistance	SSR	Bera et al., 2014
*Tifrunner × GT-C20*	Thrips, TSWV, ELS, and LLS resistance	SSR	Wang et al., 2013
Yuanza 9102 × Chico	Bacterial wilt resistance	SSR	Jiang et al., 2007
39 genotypes	*Sclerotinia minor* resistance	SSR	Chenault et al., 2009
*A. hypogaea* cv. Florunner × (*A. batizocoi* × (*A. cardenasii* × *A. diogoi*))	*Meloidogyne arenaria* resistance	RAPD	Burow et al., 1996
*A. hypogaea* × TxAg-7	*M. arenaria* resistance	RFLP	Choi et al., 1999
Interspecific cross with *A. hypogaea*	*M. arenaria* resistance	SCAR	Chu et al., 2007b
TAG 24 × ICGV 86031	Drought tolerance	SSR	Ravi et al., 2011
TAG 24 × ICGV 86031, ICGS 76 × CSMG 84-1, ICGS 44 × ICGS 76	Drought tolerance	SSR	Gautami et al., 2012
Fleur 11 × (*A. ipaensis* KG30076 × *A. duranensis* V14167)^×4^	Days to flowering, plant architecture, pod and kernel trait, yield component	SSR	Foncéka et al., 2012
Tamrun OL01 × BSS 56	Pod and kernel traits	SSR	Selvaraj et al., 2009
Zhenzhuhei × Yueyou 13	Dark purple testa color	SSR	Hong et al., 2007
TG 26 × GPBD 4	Protein content, oil content, and oil quality	SSR	Sarvamangala et al., 2011
US Peanut Minicore germplasm collection	High oleic acid content (FAD2A)	CAPS	Chu et al., 2007a
14 genotypes	High oleic acid content (FAD2B)	CAPS	Chu et al., 2009
Germplasm accessions and breeding lines	High oleic acid content (FAD2A/FAD2B)	AS-PCR	Chen et al., 2010
Germplasm accessions and breeding lines	High oleic acid content (FAD2B)	Real time-PCR	Barkley et al., 2010
Germplasm accessions and breeding lines	High oleic acid content (FAD2A)	Real time-PCR	Barkley et al., 2011

* *RAPD, Randomly Amplified Polymorphic DNA; RFLP, Restriction Fragment Length Polymorphism; CAPS, Cleaved Amplified Polymorphic Sequence; SSR, Simple Sequence Repeat; AFLP, Amplified Fragment Length Polymorphism; SCAR, Sequence Characterized Amplified Region; AS-PCR, Allele Specific Polymerase Chain Reaction*.

# *FAD, Fatty acid desaturase; TSWV, Tomato Spotted Wilt Virus; ELS, Early Leaf Spot; LLS, Late Leaf Spot*.

### Marker assisted breeding (MAB) in groundnut

**Figure 1:**
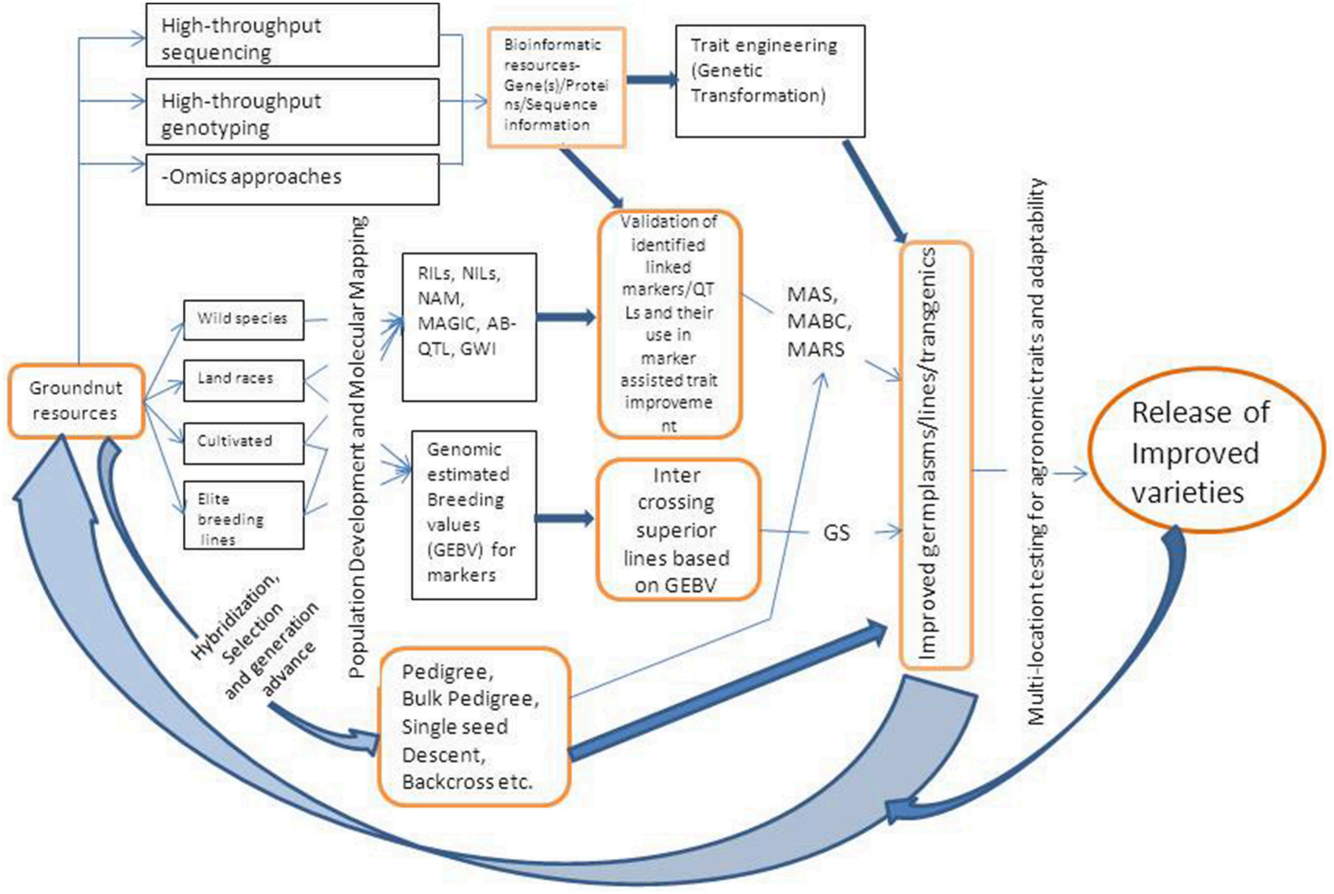
**A schematic representation of integrated breeding approach for trait improvement in groundnut**. RIL, Recombinant Inbred Line; NIL, Near Isogenic Line; NAM, Nested Association Mapping; MAGIC, Multi-parent Advanced Generation Inter-Cross Lines; AB-OTL, Advanced Backcross Quantitative Trait Loci; GWI, Genome Wide Introgression; GS, Genomic Selection; MAS, Marker Assisted Selection; MABC, Marker Assisted Backcrossing; MARS, Marker Assisted Recurrent Selection.

MAB can be practiced using either simple marker-assisted selection (MAS) approach or marker-assisted backcrossing (MABC). The selection of breeding lines in MAB requires three categories of markers (a) foreground selection, which involves using molecular markers for selecting the target gene or QTL, (b) recombinant selection, that involves selecting of backcross progenies containing the target gene, and recombination events between the target locus and linked flanking markers, and (c) background selection, where in the plants/progenies are selected based on recovery of highest proportion of recurrent parent genome (Varshney and Dubey, 2009). MAS requires foreground and recombinant markers while MABC requires all the three type of markers. Markers that are closely linked or associated with genes/QTLs for some important target traits have been identified in groundnut and are being utilized to transfer genes/QTLs to elite cultivars or to pyramid many genes either for the same trait or for different traits. A schematic diagram showing integrated breeding approach combining genotyping and phenotyping based selection of progenies is given in Figure 1.

The MAB approach was used successfully in developing NemaTAM, the first root-knot nematode resistant groundnut variety bred, and it was released for cultivation in the USA (Simpson et al., 2003a). Following the identification of markers linked to *FAD* gene alleles conferring the high oleic trait in groundnut (Chu et al., 2007a, 2009), efforts were made to pyramid nematode resistance with the high oleic trait. The nematode resistant cultivar “Tifguard” was used as recurrent female parent while Georgia-02C and Florida-07 served as donor parents for the high oleic trait to develop a variety, “Tifguard High O/L” that has resistance to nematode and high oleic trait (Chu et al., 2011). Three backcrossing were done to the recurrent parent to develop “Tifguard High O/L.” At ICRISAT, the *FAD2A* and *FAD2B* mutant alleles responsible for high oleic acid content were transferred into the elite genotypes with high oil (53–58%) and low oil content (42–50%) using MAS as well as MABC approaches. From above breeding material, several promising lines with high oleic lines and other desirable features were identified for subsequent yield evaluations (Janila et al., 2016). At ICRISAT, gene pyramiding approach was also deployed to combine early maturity with foliar fungal disease resistance and high oleic trait. In China and Japan also, MAB was deployed to breed high O/L lines in groundnut.

In a recent study at ICRISAT, the MABC approach was used successfully to combine resistance to foliar fungal diseases with early maturity. In this study, the earlier identified markers flanking the QTL controlling rust resistance were deployed (Khedikar et al., 2010; Sujay et al., 2012) leading to introgression of this QTL into three early maturing elite cultivars, namely ICGV 91114, JL 24, and TAG 24 using GPBD 4 as donor parent (Varshney et al., 2014). The same QTL also contributes 67.98% of variation of LLS resistance. Subsequently, few introgressed lines selected based on phenotype in preliminary yield trials, and disease resistance score in hot spot locations are currently in the pipeline for multi-location evaluation trials. This work is the first reported successful attempt in combining disease resistance with early maturity (Varshney et al., 2014). Besides, pyramiding genes for oil quality and foliar fungal disease resistance into 10 drought tolerant, high oil, recently released cultivar backgrounds is also in progress at ICRISAT. The next decade may probably witness a good number of groundnut varieties developed through integrated molecular breeding approaches.

### Genomic tools for harnessing natural variation in groundnut

The availability of molecular markers has played key role in increased utilization of wild species and land races in breeding programs, as it helps in reducing the linkage disequilibrium that is frequently associated with lines developed through wide hybridization. In groundnut, tapping of wild alleles into the cultivated gene pool is restricted by (a) crossing barriers imposed by ploidy differences between wild and cultivated groundnut (b) association of favorable alleles with unfavorable alleles, a process referred to as linkage drag, and (c) non-availability of efficient tools to identify hybrids and track introgressed chromosomal segments. Systematic introgression of entire genome of a wild species into cultivated background is now possible by use of molecular markers, which is referred to as genome-wide introgression (GWI). Following this approach, it is possible to develop chromosome segment substitution lines (CSSLs) and advanced back-cross QTL (AB-QTL) mapping populations which enable tapping of new alleles from wild species. GWI and AB-QTL have been used to generate variability in cultivated groundnut. GWI facilitates the transfer of a small genomic region from the wild donor parent into the homozygous genetic background of elite parent. At ICRISAT, by tracking the wild genome introgression through genotyping, AB-QTL population is under development by crossing a synthetic amphidiploid, ISATGR 184(5) with popular cultivar, Tifrunner (Accession number: ICG 9937). ISATGR 184 (5) is derived from a cross between two diploid wild progenitor species, *A. ipaensis* (Accession number, ICG 8206), and *A. duranensis* (Accession number, ICG 8123). AB-QTL, an approach that facilitates simultaneous discovery and transfer of QTLs into elite genotypes (Tanksley and Nelson, 1996), was recently utilized by Foncéka et al. (2012) in groundnut and involved a cross between the wild synthetic amphidiploid of (*A. ipaensis* × *A. duranensis*)^4X^ and the cultivated Fleur 11 variety. The CSSLs (Foncéka et al., 2009) and AB populations facilitate characterization of different segments of wild species contributing for resistance to foliar diseases and/or any other desirable trait. Once these different segments and their roles are determined, it is then possible to track them along the back-crosses using molecular markers for use in breeding programs. From AB-QTL populations developed earlier at ICRISAT and foliar fungal disease resistant lines were identified in disease nurseries that may server as potential sources to broaden the genetic base of foliar fungal disease resistance after validating with known QTLs. At present, GPBD 4 is used as a source of resistance to LLS and rust governed by two major QTLs and there is need to identify new genomic regions from new sources to achieve durable resistance through gene pyramiding approach.

There has been a speedy developments in development of genomic resources and genomic tools for breeding (see (Pandey et al., 2012; Varshney et al., 2013; Janila et al., 2014), http://www.peanutbioscience.com/). Simple sequence repeat (SSR) markers are still the choice of markers for breeders but their number were very less till now i.e., ~2500. Meanwhile, ICRISAT in collaboration with DArT Pty Ltd., Australia has developed diversity arrays technology (DArT arrays) with 15,360 features (see Pandey et al., 2014) and Kompetitive Allele Specific PCR (KASP) assays for 90 SNPs (Khera et al., 2013). The above resources were used for construction of several genetic maps, linkage analysis, trait mapping, and molecular breeding (see Pandey et al., 2012; Varshney et al., 2013; Janila et al., 2014, http://www.peanutbioscience.com/). However, sequencing and resequencing data generated for draft genome assembly will now be used for development of several 100 SSRs and millions of SNP evenly distributed in the peanut genome. The availability of huge SNP resources has led to initiate the development of 60 K SNP chip by ICRISAT in collaboration with University of Georgia (UGA), USA using Affymetrix SNP platform for accelerating genetics and breeding applications in these legume crops.

### Functional genomics tools for gene discovery in *Arachis*

The use of functional genomics and biotechnological techniques serve as important tools as they enable the discovery and characterization of genes of agronomic importance through deep analysis of transcriptome, and their direct transference to chosen cultivars by plant transformation (Brasileiro et al., 2014). Genes encoding storage proteins and fatty acid metabolic enzymes, genes that are differentially expressed in response to pathogen stress, genes involved in oil metabolism etc., have been identified and cloned in groundnut through EST sequencing and are being utilized in groundnut improvement. For example, to improve oil stability in groundnut a *FAD2* gene RNAi construct was transformed into groundnut (Zhang et al., 2007; Huang et al., 2008) and the resulting transgenic plant showed an increased O/L ratio (Huang et al., 2008). Similar RNAi technique was used to repress the accumulation of major allergens Arah 1 and Arah 2 in groundnut (Dodo et al., 2008). Functional genomic resources such as expressed sequence tags (ESTs), have been used to understand temporal and spatial gene expression patterns, for development of gene based markers and maps, transcript profiling to identify candidate genes involved in expression of traits of interest, and the identification of transcription changes during biotic and abiotic stresses. The Microarray technology was used to study expression patterns of several genes from diverse tissues such as groundnut seed, leaves, stems, roots, flowers, and gynophores (Bi et al., 2010), and to analyze transcript levels in different tissues and organs in order to identify pod specific groundnut genes and correlated to seed storage proteins, desiccation, oil production, and cell defense (Payton et al., 2009). Another important application may be to understand molecular mechanisms governing host-pathogen interactions that include pathogen-associated molecular pattern (PAMP), pathogen triggered immunity (PTI), and effector triggered immunity (ETI) in plants. Transcriptome resources generated through microarray or next generation sequencing (NGS) tools are of immense value in species where genome sequence is not yet available (Varshney et al., 2009).

EST sequencing allows identifying plant genes that are preferentially expressed in specific organs or plant tissues at a particular time. Using the technique of microarray, it is possible to detect the spatial and temporal distribution of gene expression among tissues and genotypes. This potential has been exploited in *Arachis* to generate ESTs for specific traits such as drought (Jain et al., 2001), tomato spotted wilt virus and leaf spot disease resistance (Guo et al., 2009), *Aspergillus* infection and aflatoxin contamination (Guo et al., 2008); tissue specific ESTs such as in root, leaf, seedlings, developing pods etc. (Nagy et al., 2010; Koilkonda et al., 2012); stage specific ESTs such as different developmental stages (Song et al., 2010), and seed development (Bi et al., 2010). ESTs from wild species have also been developed (Luo et al., 2005a,b). So far, the EST based approach has resulted in the isolation of 254,541 ESTs (available in public database) from *A. hypogaea, A. duranensis, A. ipaensis, A. stenosprema*, and *A. magna* (Brasileiro et al., 2014).

### Emerging genomic technologies and groundnut genome sequence

Genomic selection (GS) is another emerging technique for crop improvement. This technique relies on the identification of superior lines with higher breeding value i.e., genomic estimated breeding value (GEBV) in segregating breeding populations based on genome-wide marker profile data. To estimate GEBV, a training population (TP) comprising of elite breeding lines for which multiple season phenotyping data on agronomically important traits are available across environments is used. The GEBVs are then used for selecting the appropriate parents and using them in crossing programs to develop candidate population (CP). GS is now the preferred method of choice over MABC and marker-assisted recurrent selection (MARS) for improving complex traits such as yield under drought condition. At ICRISAT, to utilize GS approach in groundnut improvement, a TP has been developed that includes about 340 advanced breeding lines for which historical data on their performance have already been compiled and are on the process of being evaluated in multi-location trials for important agronomic, quality, and biotic and abiotic stress tolerance/resistance traits.

The availability of emerging genomic technologies especially NGS has enabled the generation of a lot of sequence data for a number of plant species (Varshney and Dubey, 2009). NGS technologies are powerful tools for functional genomic studies as it enables comprehensive transcriptome analysis of induced changes in gene expression, allows the prediction of the roles and interactions of individual or correlated genes, and helps the elucidation of more complex signaling pathways activated in response to external stimuli. These techniques have also made available limited sequence information, those having large genome size, and those exhibiting polyploidy. The “International Peanut Genome Initiative (IPGI, http://www.peanutbioscience.com/peanutgenomeinitiative.html)” has decoded the draft genome for both the diploid progenitors i.e., A—(*Arachis duranensis*) and B—genome (*A. ipaensis*) with 1.1 and 1.38 Gb genome size, respectively. In addition to above genomes, another initiative called “Diploid Progenitor Peanut A-genome Sequencing Consortium (DPPAGSC)” has also completed genome sequencing for another accession of *A. duranensis* with 1.07 Gb genome size (see Varshney, 2016). The recent developments also made available opportunities to generate high throughput genotyping data using NGS-based genotyping platforms. As a result, the first ever SNP genetic linkage map for cultivated groundnut was constructed with 1621 SNPs and 64 SSR loci (Zhou et al., 2014). The majority of the published and unpublished information on genomic resources such as genome sequence, transcriptome sequences, and trait mapping information have been made available at the http://www.peanutbioscience.com. It is expected that the availability of draft genome sequence along with extensive genomic and transcriptome information will enable deployment of modern genotyping approaches such as genotyping-by-sequencing (GBS) at cheaper costs.

## Transgenics

Transgenic approach has great potential in groundnut improvement as it is not limited by ploidy and crossability barriers, and it is virtually possible to transfer genes across taxa. However, its use has been hindered by public resistance to GMO (Genetically Modified Organism) food crops, difficulties for plant regeneration by tissue culture techniques and selection of transgenic events (Holbrook et al., 2011; Brasileiro et al., 2014). Notwithstanding the limitations, transgenic groundnuts lines expressing genes that modulate different traits such as resistance to virus, insect and fungus, drought tolerance and grain quality have been developed by several research groups, particularly in United states, China and India which are currently under evaluation at different containment levels: *in vitro*, greenhouse, and field conditions (Ozias-Akins and Gill, 2001; Holbrook et al., 2011). The recent progress achieved in *Arachis* genetic transformation has been reviewed by Holbrook et al. (2011) and Brasileiro et al. (2014).

## Summary

Conventional breeding has contributed to improvement of agronomic, quality and stress tolerance traits in groundnut by using existing variability in cultivated gene pool, however there is immense scope to improve breeding efficiency (time and resources), accuracy, and enhancing genetic gain with use of genomic tools. Use of genomic tools in breeding program results in enhanced rate of genetic gain for target traits and also enable to combine multiple traits. Besides, molecular markers also enable tapping of desirable alleles from wild species without the burden of linkage disequilibrium. The development of molecular markers liked to target traits is a key step in integrating genomics with groundnut breeding. Construction of molecular marker linkage maps in groundnut and identification of markers associated to gene/QTL(s) for important target traits paved the way for deployment of genomic tools in breeding program. With the identification of markers linked to gene/QTL(s), MAS is now common and moving toward gene pyramiding for combining multiple traits. For example, markers linked to LLS and rust resistance, and markers for high oleic acid content are being used to introgress these traits into short duration, high oil containing drought tolerant cultivars. Different type of populations such as GWI, AB-QTL, MAGIC, NAM, RILs, NILs, etc., are now available to map QTLs and carry out association studies in groundnut. Emerging genomics technologies such as NGS and high throughput marker genotyping using SNPs have enabled the generation of a lot of sequence data for groundnut. The draft genome sequences for the two diploid progenitor species are now available in groundnut. These sequences are expected to aid in identification of genes in the cultivated species, once the draft genome sequence of *A. hypogaea* L. becomes available. Groundnut breeders in developed countries have made better use of molecular breeding tools than developing countries, which is in part due to inadequate infrastructure, high genotyping costs, and inadequate human capacities in the latter. But the accessibility and utilization of integrated breeding (e.g., use of MAB) in developing countries is expected to expand with improved affordability of using genomic tools (e.g., genotyping) with advances in molecular techniques.

## Author contributions

All authors listed, have made substantial, direct and intellectual contribution to the work, and approved it for publication.

### Conflict of interest statement

The authors declare that the research was conducted in the absence of any commercial or financial relationships that could be construed as a potential conflict of interest.
